# Bridging the gap between public health, academia and policy

**DOI:** 10.1136/bmjgh-2025-019587

**Published:** 2026-06-22

**Authors:** Paula Christen, Jeanette Dawa, Omar Ahmed, Florence Akinyi, Bonventure Ameyo Masakhwe, Daniel Biftu Bekalo, Cosmas Biwot, Arani B Bosire, Giovanni Charles, Veronicah Gathoni, Katy A M Gaythorpe, Githinji Geoffrey, Salmon Gowa, Fathiyah Said Hamumy, Hannah Kagiri, George Kamundia, Stephanie Kamunya, Pius Kariuki, Lydiah Khalayi, Ivy Kombe, Purity Kwamboka, Paul Liétar, Noel Likalamu, Brian Lugonzo, Vincent Mariita, Augustine Masinde, Mumbua Mbithi, Ruth McCabe, Fred Nyamitago Monari, Francis Ondicho Motiri, Antony M Muchiri, Annah Mudamba, Peter Waweru Mugo, Nyamai Mutono, Moses Muriithi, Ryan Musasia, Mumbua Mutunga, Waithera Mwangi, John Nalwa, Kibet Immanuel Ngeno, Jackline Mosinya Nyaberi, Peninna Mwongeli Nzoka, John Ojal, Tabitha Okech, Emmanuel Okunga, Evans Omondi, Joseph Osoro, Preston Osoro, Amika Patel, Stanley Sayianka, Triza Shigoli, Catherine Silali, Martin M Thendu, Sabine L van Elsland, Kariithi Anne Wanjiku, Philip Muchemi, Lilith K Whittles, Peter Winskill, Loice Achieng Ombajo, S M Thumbi, Oliver J Watson

**Affiliations:** 1 Centre for Epidemiological Modelling and Analysis, University of Nairobi, Nairobi, Kenya; 2 Medical Research Council Centre for Global Infectious Disease Analysis, Imperial College London School of Public Health, London, UK; 3 University of Nairobi, Nairobi, Kenya; 4 Mombasa County Government, Mombasa, Kenya; 5 Division of Disease Surveillance and Response, Kenya Ministry of Health, Nairobi, Kenya; 6 Turkana County, Kenya Ministry of Health, Turkana, Kenya; 7 Jomo Kenyatta University of Agriculture and Technology, Nairobi, Kenya; 8 Kenya National Public Health Institute (KNPHI), Nairobi, Kenya; 9 Qhala, Nairobi, Kenya; 10 Machini Tech, Nairobi, Kenya; 11 National Vaccines and Immunization Program, Kenya Ministry of Health, Nairobi, Kenya; 12 Health Sector Monitoring and Evaluation, Kenya Ministry of Health, Nairobi, Kenya; 13 IntelliSOFT consulting, Nairobi, Kenya; 14 Kenya Medical Research Institute (KEMRI) Wellcome Trust Research Programme, Kilifi, Kenya; 15 Veterinary Epidemiology and Economics Section (VEES), Directorate Veterinary Services, Nairobi, Kenya; 16 Kisii University, Kisii, Kenya; 17 Division of National Laboratory Services (DNLS), Kenya Ministry of Health, Nairobi, Kenya; 18 Digital Health, Policy and Informatics, Kenya Ministry of Health, Nairobi, Kenya; 19 Washington State University Paul G Allen School for Global Health, Pullman, Washington, USA; 20 BCL, Nairobi, Kenya; 21 20Four7Va, Nairobi, Kenya; 22 African Population and Health Research Center, Nairobi, Kenya; 23 Institute of Mathematical Sciences, Strathmore University, Nairobi, Kenya; 24 Finesse, Nairobi, Kenya; 25 The Nairobi West Hospital Ltd, Nairobi, Kenya; 26 The University of Edinburgh Institute of Immunology and Infection Research, Edinburgh, UK

**Keywords:** Public Health

## Abstract

The use of advanced analytics in public health policy remains hindered by a disconnect between researchers, policymakers and technical experts. Bridging this gap requires intentional knowledge translation strategies that facilitate interdisciplinary collaboration and real-world application of research findings. Hackathons, which bring together diverse stakeholders in a time-bound, solution-oriented format, offer an approach to address this challenge. In January 2025, the MRC Centre for Global Infectious Disease Analysis and the Centre for Epidemiological Modelling and Analysis at the University of Nairobi organised the *Bridging the Gap Hackathon*, designed to strengthen collaboration between academia, policy and public health practitioners in Kenya. The hackathon convened researchers, software engineers and policymakers to co-develop data-driven tools to tackle public health challenges identified by Kenya’s Ministry of Health and the Directorate of Veterinary Services. Over five days and using a structured multi-stage process, six interdisciplinary teams developed prototype solutions to improve outbreak surveillance, vaccine deployment, data quality monitoring and health workforce estimation. This paper reflects on the hackathon’s structure, participant experiences and project outcomes, highlighting key lessons for future knowledge translation initiatives. Our findings suggest that hackathons can serve as effective platforms for accelerating interdisciplinary research impact, fostering engagement between policymakers and researchers and promoting the development of solutions to public health issues.

**SUMMARY BOX**

- Hackathons offer a structured and replicable approach to intentional knowledge translation, enabling researchers, policymakers and technical experts to co-develop solutions that address real public health priorities.
- Continuous engagement of decision-makers throughout the hackathon ensured that prototypes were grounded in practical realities, increasing their relevance and potential for future uptake.
- Participant feedback highlighted the value of structured facilitation, daily reflection and team diversity in fostering collaboration, skill-building and problem-solving capacity.
- The Bridging the Gap Hackathon demonstrates that time-bound, collaborative formats can strengthen research–policy linkages and offer a promising model for accelerating locally relevant public health innovation in low- and middle-income settings.

## Introduction

The integration of advanced analytics into public health policy and programmes is often hindered by a disconnect between researchers and policymakers. This gap, commonly referred to as the ‘knowledge-to-action’ gap, underscores the necessity for robust and intentional knowledge translation strategies that facilitate the development and application of research findings in policy and practice settings.^1 2^

Studies have highlighted that, despite the availability of pertinent research, the absence of established knowledge translation frameworks impedes timely and effective policy formulation.^3^ Additionally, systemic barriers such as inadequate communication channels, misaligned priorities and incentives^4^ and limited policymaker engagement with research institutions exacerbate this issue.^5 6^ Public health emergencies (eg, the COVID-19 pandemic^7^), as well as general public health policy development (eg, policy and programmes working towards the 2030 Agenda for Sustainable Development^8^), revealed the importance of building trust and collaboration between researchers, policymakers and the public to foster evidence-informed decision-making. A study on policy decision-making during the COVID-19 pandemic in low- and middle-income countries emphasised factors such as solid relationships and open communication between policymakers and research teams, the credibility of researchers, and co-creation of policy questions, models and their insights as critically important for knowledge translation.^9^

Moreover, during the COVID-19 pandemic, there were significant contributions from individuals and groups beyond traditional public health disciplines. Notably, professionals from fields such as macroeconomics and artificial intelligence (AI) played pivotal roles in data analysis and predictive modelling, thereby informing policy decisions. For instance, the World Mortality Dataset was developed by a macroeconomist and AI researcher pair, which provided an invaluable database and tool that was relied on by the WHO in their estimates of excess mortality associated with the COVID-19 pandemic.^10 11^ This interdisciplinary collaboration underscores the value of diverse expertise in addressing complex public health challenges.^12^ Furthermore, citizen science and crowdsourcing made significant contributions. For example, developers mapped the daily trajectory of COVID-19 and tracked its progression in different parts of Kerala, India, on an online platform to overcome the limitations of unstructured data released by the government and generated open and reusable datasets for analysis and visualisation.^13^

This practice paper seeks to demonstrate how hackathons can serve as an intentional knowledge-translation mechanism that bridges research, policy and practice in public health. Rather than evaluating the long-term impact of the resulting tools, our objective is to reflect on the process of the ‘Bridging the Gap Hackathon’ (BTGH) as a means of facilitating interdisciplinary collaboration, capacity building and rapid co-creation of data-driven solutions. By doing so, we aim to legitimise hackathons as a structured and replicable approach to accelerating research uptake and policy relevance in low- and middle-income country settings.

## Components of a public health knowledge translation hackathon

Stakeholder engagement initiatives aiming to enhance advanced analytics, visualisations and software tools (hereafter referred to as ‘technical resources’) for use in health policy operate with a dual focus: generating ‘better science’ and facilitating ‘better decisions’.^14^ By incorporating stakeholder input, technical resources can be tailored to address local concerns and challenges, increasing their utility and relevance. Engagement in the process of developing technical resources can help parameterise technical resources with realistic field data^15^ and ensure that the ‘needs’ and ‘preferences’ of stakeholders embedded in local communities are represented.^16^ As technical resources are attuned to local contexts, their quality and outputs can improve.^14^

Further, the value of stakeholder engagement in developing technical resources lies in its potential to enhance the capacity of stakeholders to respond to the challenge at hand. Technical resources can function as ‘tools’ to help build a shared understanding or ‘common “mental map” of the health problem’,^17^ serving as a ‘rallying point’ around which stakeholders can gather.^18 19^ This shared understanding can, for example, be used by health planners as a catalyst for convening diverse stakeholders to think about their strategic directions and policy priorities, ultimately aligning actions and fostering more informed decision-making.^20^ Engagement can also foster ownership and trust, enhancing the relevance of research, mutual learning, transparency and understanding of research processes.^21^

Hackathons have emerged as a novel approach to fostering stakeholder engagement (box 1).^22 23^ These time-bound, collaborative events convene diverse participants to co-create solutions addressing specific health challenges. By facilitating interdisciplinary engagement, hackathons serve as dynamic platforms for intentional knowledge creation and translation, translating research insights into practical applications and informing health policy and programme development. This format can foster a collaborative environment where all engaged parties find value and relevance, from the conceptualisation of the challenge to the presentation and use of the technical resource. Leveraging hackathons could enhance the synergy between research, practice and policy in Kenya and globally, thereby strengthening the health system’s responsiveness to emerging challenges.^24^

Box 1 **What is a hackathon?**

**Definition of a hackathon**

Hackathons are structured events where participants work intensively on a specific problem over a fixed period, typically ranging from hours to days. Originally emerging in the context of coding competitions, hackathons have expanded across disciplines, including policy, healthcare and law, as a means of driving creativity, rapid problem-solving and interdisciplinary collaboration. These events provide a focused environment where participants can generate solutions without the usual distractions of academic or professional work. By assembling individuals from diverse backgrounds, such events facilitate ideation and innovation, allowing participants to develop novel approaches that might not emerge through traditional research processes.^25^

**Benefits of a hackathon for public health policy**

Beyond immediate problem-solving, hackathons offer broader benefits for participants and their fields of study. They empower individuals to engage with challenges they are passionate about, gain practical experience in collaborative problem-solving, and refine technical and strategic skills. In public health, hackathons can highlight pressing issues and harness the expertise of diverse contributors to develop actionable solutions, particularly in contexts where research capacity is constrained.^26^ By fostering a sense of community, these events encourage continued engagement with the ideas generated, supporting long-term collaboration and sustained innovation beyond the hackathon itself.

**Comparison with other participatory approaches**

Hackathons share key features with other participatory knowledge translation approaches used in low- and middle-income countries, such as designathons, participatory action research (PAR) and policy labs. While designathons typically follow a structured, design-thinking process with planned follow-up phases,^25^ PAR and policy labs unfold over longer timelines to support deep community or institutional engagement.^27 28^ In contrast, hackathons offer a rapid, time-bounded format that fosters cross-sector collaboration and delivers early-stage solutions quickly. Their distinctive value lies in convening diverse participants, including end-users and policymakers, in an intensive co-production process that generates momentum and actionable outputs with potential for further development.

Hackathons have proven effective in diverse domains such as biomedical innovation, entrepreneurship and civic technology. For instance, MIT’s ‘Hacking Medicine’ series has led to multiple start-ups and validated prototypes adopted by healthcare providers, while hackathons organised under the WHO Health Innovation Exchange have produced open-source tools for surveillance and digital health delivery in low-resource settings. Similar formats in civic technology, such as the NASA Space Apps Challenge and GovHack, have generated data visualisation platforms later integrated into government decision-making systems. These examples demonstrate the potential of hackathons to translate ideas into actionable products and policies, a principle we sought to adapt to the public-health analytics context in Kenya.

## From public health priorities to research-driven tools: an adapted hackathon

In January 2025, the MRC Centre for Global Infectious Disease Analysis (MRC-GIDA) and the Centre for Epidemiological Modelling and Analysis (CEMA) at the University of Nairobi organised a hackathon focusing on bridging the gap between public health research and policy in Kenya. The BTGH used an adapted hackathon structure to meet three objectives:

- Addressing present challenges identified by officials from Kenya’s Ministry of Health (MoH) and Ministry of Agriculture and Livestock Development with data-driven solutions.
- Bringing individuals with different perspectives and expertise together to tackle these identified challenges.
- Build a foundation for longer-term research partnerships between the two centres for epidemiological analysis and modelling of infectious diseases.

**Figure 1:**
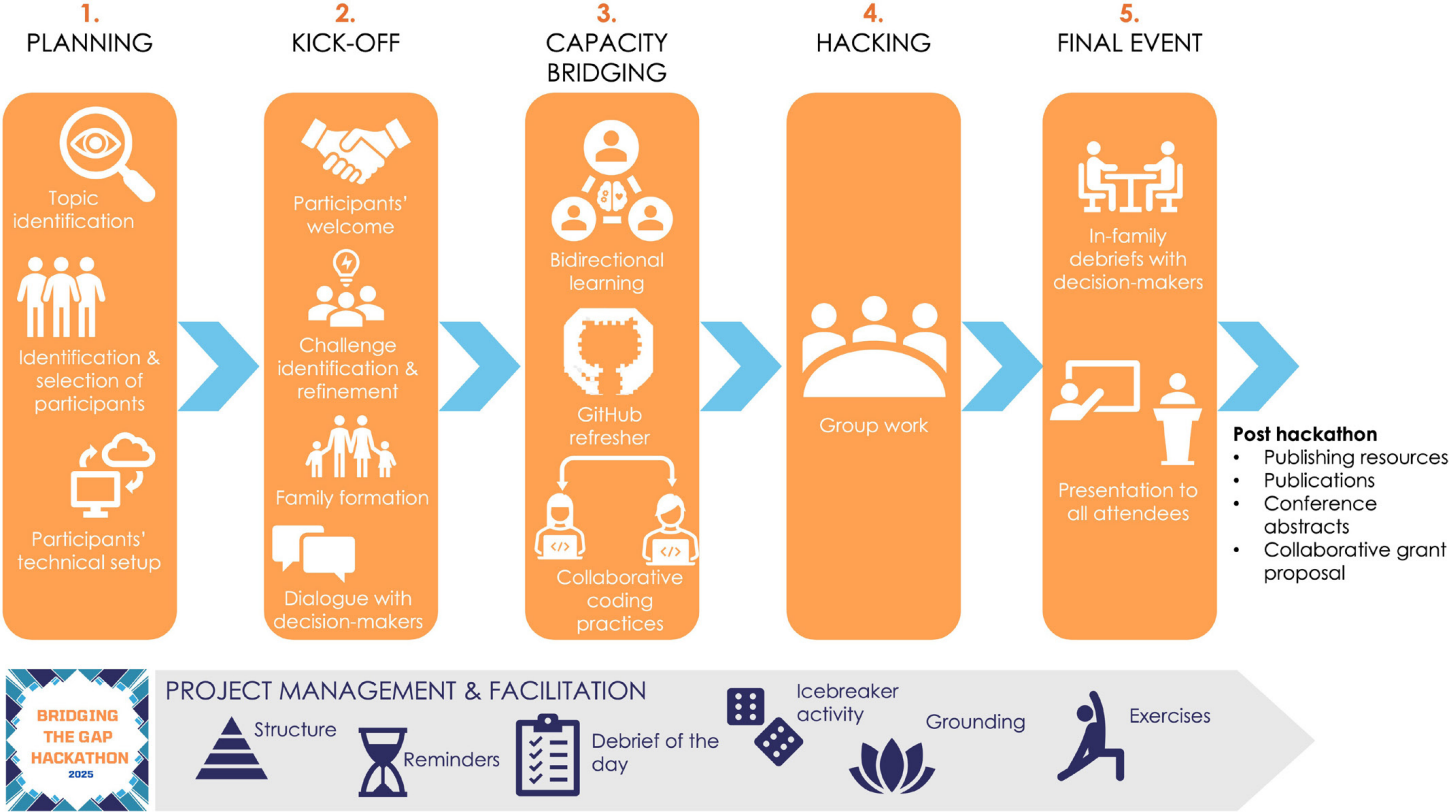
Flow diagram of the Bridging the Gap Hackathon stages.

The hackathon comprised 10 stages: (1) topic identification, (2) identification and selection of participants, (3) participants’ technical setup, (4) participant welcome, (5) challenge identification and refinement, (6) family formation, (7) dialogue with decision-makers, (8) GitHub refresher, (9) family work, (10) discussion with and dissemination to decision-makers (figure 1).

- *Topic identification*. In the pre-work stage, CEMA engaged in conversations with public health decision-makers from the MoH and the Directorate of Veterinary Services of the Ministry of Agriculture and Livestock Development to identify challenge areas for the hackathon. These conversations resulted in six challenge areas, each of which featured a request for a solution to a current priority public health issue.
- *Identification and selection of participants*. The attendees for the hackathon were identified in three ways:
  1. Open application for Kenyan researchers that included software engineers, data scientists and modellers (hackers).
  2. Invited public health decision-makers engaged in one of the six challenges identified a priori (decision-makers).
  3. Invited academic staff from Imperial College London, CEMA and Kenya Medical Research Institute-Wellcome Trust Programme with aligned research interests with the six challenges identified a priori (facilitators).
    The hackathon was advertised on social media channels (LinkedIn and X), and to the CEMA and University of Nairobi networks via email. Applicants were requested to fill out a survey (https://forms.gle/YUfnKsCfhmjMMQ2p6) to gather information on their demographic, professional and technical background and evidence of coding proficiency (demonstrated through links to GitHub profiles) to ensure a diverse participant pool. Specific questions focused on applicants’ expertise, familiarity with R programming, and their capacity to contribute to interdisciplinary problem-solving. Additionally, applicants were asked to share which topics and challenges they believed should be addressed during the hackathon.
    Responses informed purposeful participant selection and allocation of limited travel funding, emphasising inclusivity and alignment with the hackathon’s objectives.
- *Participants’ technical setup*. Participants were reminded to have the necessary software installed on their devices and to familiarise themselves with GitHub. Supporting self-paced training material (tutorials and learning resources) was linked on the hackathon’s website (https://cema-mrc-hackathon.github.io/preread.html). To facilitate collaborative coding and anticipate the need for private GitHub repositories if sensitive data was required for hackathon challenges, all participants were added to a GitHub organisation (https://github.com/CEMA-MRC-Hackathon).
- *Participant welcome*. During the participant welcome stage, a presentation on what to expect during the course of the hackathon was given, and grounding activities were conducted to establish participant norms of communication and build rapport among attendees. A facilitated icebreaker exercise, Jenga with informal question prompts, created an open and inclusive environment, fostering collaboration from the outset (online supplemental figure 1). Participants were randomly assigned to groups, encouraging a relaxed atmosphere where they could comfortably acclimate to the setting. This approach helped participants loosen up, express themselves more freely, and engage in conversations with one another.
- *Challenge refinement*. Participants were introduced to the pre-identified topics by giving everyone 30 min to read through a document outlining each of the topics. Participants were then encouraged to share input through post-it note exercises and brainstorming sessions to identify key challenges around the topic. This phase also welcomed new topic ideas, allowing participants to expand the scope of the hackathon through their unique perspectives. Thereby, all participants had time to engage with each of the topics, contribute to their refinement, and enhance creative thinking. Then, individuals were asked to use Post-its again to share about their research or technical/software skills, which challenges they would like to work on, their first name and initial of last name, for example, Oliver W, and their affiliation, for example, CEMA.
  All input on the challenges on Post-it notes was digitised and analysed semantically. This process was AI-assisted using ChatGPT-4o to allow for faster processing and seamless continuation of the hackathon. All Post-it notes on participants’ skills and challenge preferences were also digitised in tabular format (online supplemental figure 2).
- *Family formations*. The topics with challenges were presented with examples of which analyses could be of policy and programme relevance (table 1). Preliminary groups, which we referred to as families, were formed based on participants’ challenge preferences, skills and affiliations. Each family had to include at least one member affiliated with CEMA so that the work could be continued beyond the hackathon. Further, the family formation stage involved skills sharing and matching, ensuring that interdisciplinary families were composed of individuals with complementary expertise and that individuals’ challenge preferences could be taken into account. This facilitated the creation of robust families equipped to tackle multifaceted public health challenges.
- *Dialogue with decision-makers*. Decision-makers were invited to join the designated challenge families to provide context for the issue at hand, share their perspectives, ask questions, assess data availability, clarify the resources hackers could have access to, define outcome measures, discuss any assumptions and set expectations.
- *GitHub refresher*. To ensure everyone had at least foundational knowledge of GitHub, day 2 started with a 2-hour GitHub refresher provided by OJW and PL. This covered an overview of Git as a version control system, explaining its purpose in tracking file changes over time and introducing needed terminology (repositories, commits, push/pull request). Finally, this covered how to use GitHub for coding collaboratively, before providing an overview of how to create SSH keys needed to work on private GitHub repositories, as well as how to integrate Git/GitHub with common IDEs such as RStudio.
- *Family work*. Family work formed the core of the hackathon, providing participants with collaborative coding training, facilitation guidance and project work time. These activities were supported by several recurring practices implemented throughout the week (box 2). Daily goal-sharing sessions and project-planning activities were facilitated to maintain focus and momentum. Mentors were available throughout, offering technical expertise but also contributing to the solution development as participants. Families were encouraged to continuously integrate decision-makers while refining their solutions to ensure alignment with their preferences and needs.

**Table 1:** Challenges identified during the brainstorming session by topic

Topic	Challenges
Cold chain capacity monitoring	Develop Internet of Things (IoT)-based cold chain monitoring sensors. Example: ‘Add an indicator in KHIS for monthly reporting of cold chain capacity and equipment functionality’ Create an app for real-time temperature alerts. Example: ‘Enable automated temperature monitoring with real-time notifications for deviations’ Design an automated cold chain capacity planner. Example: ‘Develop predictive models to estimate cold chain needs based on vaccine demand trends’
Outbreak alert thresholds	Develop a user-friendly data visualisation dashboard. Example: ‘Create visuals to track outbreak alerts and response timelines’ Automate data validation processes. Example: ‘Integrate systems to validate alert thresholds against historical data’ Implement a centralised database for data integration. Example: ‘Develop a unified platform to consolidate outbreak reports from multiple regions’
Data quality validation	Create a data quality scoring system. Example: ‘Automate data validation checks for anomalies in health records’ Design a dashboard for tracking data anomalies. Example: ‘Develop real-time analytics for monitoring data quality metrics’ Automate alerts for outliers in data submissions. Example: ‘Enable system-generated notifications for inconsistencies in vaccination data’
Malaria vaccine deployment	Create a vaccine deployment tracker. Example: ‘Track malaria vaccine rollout progress at the sub-county level’ Develop GIS tools to map vaccine coverage. Example: ‘Visualise vaccine distribution areas to identify coverage gaps’ Design an algorithm to optimise vaccine distribution. Example: ‘Simulate distribution scenarios to ensure equitable vaccine access’
Animal surveillance data integration	Develop spillover risk mapping tools. Example: ‘Map high-risk areas for zoonotic disease spillovers based on surveillance data’ Automate surveillance reporting systems. Example: ‘Streamline submission of surveillance data to improve reporting efficiency’ Implement interoperability features between animal and human surveillance. Example: ‘Integrate animal health data into human surveillance platforms for a coordinated response’
Workforce estimation	Develop a workforce simulation tool. Example: ‘Model staffing requirements for vaccination campaigns’ Create a training needs assessment platform. Example: ‘Identify skill gaps and design targeted training programmes for health workers’ Design a dashboard to monitor workforce metrics. Example: ‘Track regional workforce deployment and utilisation in real-time’

GIS, Geographic Information System; KHIS, Kenya Health Information System.

Box 2 **Recurring tasks and hackathon guidelines**

- Debrief the day: at the end of each day, in a 30-minute debrief time, each family was encouraged to reflect on question prompts.
- All participants, except decision-makers, were encouraged to contribute to software development through coding. This was enabled by asking families to sequentially commit on GitHub by different family members.
- Participants were asked to share immediate feedback throughout the hackathon via an anonymous Google Survey. Thereby, the organisers hoped to be able to continuously improve the hackathon, sharing updates and responses to the feedback in real-time at the start of each day.
- Various forms of ‘Icebreaker Jenga’ were played during the course of the hackathon, which enabled relationship-building and fostered creative thinking in non-coding times.

- *Discussion with and dissemination to decision-makers*. As part of the final stage, families dedicated half a day to preparing and delivering presentations to decision-makers. The families presented their solutions through live demonstrations to decision-makers, providing an interactive opportunity for policymakers to engage directly with the software. Decision-makers could ask questions, provide feedback and suggest refinements to align the solutions more closely with their needs.
  - Following these interactive sessions, families presented their work to the entire audience, fostering a broader discussion on the policy value of their solutions. These presentations outlined key aspects of their projects, including the background (context, rationale and objectives), a technical overview, and a live or semi-live demonstration of their tool. Additionally, families discussed the next steps, highlighting features that were not covered during the week and potential enhancements. A crucial component of this session was outlining a maintenance plan, specifying who could take the work forward at CEMA. As part of these presentations, decision-makers were asked to reflect on the potential value of the projects for their decision-making space.
  - Families were also encouraged to consider opportunities for broader dissemination. This included identifying relevant conferences - both Kenya-based and international - where they could submit abstracts to present their work. A list of potential venues was provided on the conference website (https://cema-mrc-hackathon.github.io/conference.html). Beyond conference presentations, participants were urged to explore further avenues for dissemination, such as continuing the collaboration, publishing in peer-reviewed journals and seeking funding to advance their projects.

## Evaluation of the BTGH: outcomes, reflections, recommendations

### Outcomes

The outcomes of the hackathon are presented here with a focus on the nature and scope of the prototype solutions generated, rather than on their long-term implementation or policy impact. As the hackathon concluded in early 2025, follow-up evaluation of adoption, scalability and sustainability is planned as part of ongoing collaborations between CEMA, Imperial College London and MoH. The intent of this section is therefore to document the immediate outputs of the hackathon - illustrating what was produced, how predefined challenges were addressed and how these prototypes reflect the potential of hackathons as platforms for applied knowledge translation.

Across ten structured stages, the BTGH produced six prototype solutions addressing key policy challenges, while engaging a diverse participant base. For the open application, 282 applications were received (226 Kenya-based), covering 15 out of 47 counties in Kenya, with 41.1% of applicants from Nairobi. The 282 applications spanned a variety of disciplines: 36% academics and researchers (participants from various Kenyan institutions, not limited to CEMA or MRC-GIDA at Imperial College London who are involved in infectious disease modelling and public health data analysis), 44% software developers and technical experts (individuals proficient in R programming, version control using Git and GitHub, programming, software development, epidemiological modelling, quantitative analysis, statistical analysis and related subjects) and 20% health decision-makers and providers (MoH officials and public health specialists who provide insights based on real-world public health needs and ensure developed tools address practical challenges).

Participants included hackers, decision-makers and facilitators. 23 hackers were accepted and attended the event, of whom eight were principally software development focused and 15 with a background in public health research, statistics, mathematics and geostatistics, among others. The 23 hackers were joined by 14 decision-makers and 16 facilitators (online supplemental figure 3). Overall, participants were 60.4% (32/53) male and 83% (44/53) Kenyan.

Six families emerged from BTGH, tackling a wide array of public health issues (table 2). Each area focused on a known public health need as identified by a present decision-maker.

**Table 2:** Challenges addressed during the hackathon

Topic	Objective
Cold chain capacity monitoring	Develop an interactive dashboard for monitoring and reporting cold chain storage status, for example, cold chain capacity, space utilisation, available capacity and facility locations with attributes
Outbreak alert thresholds	Effective visualisation of measles outbreaks and response strategies through a scenario planning tool for measles vaccinations at the sub-county level
Data quality validation	Develop automated processes to identify and validate data anomalies in vaccine administration records
Malaria vaccine deployment	Assess the impact of scaling ITNs, IRS, SMC and the malaria vaccine (RTS,S/AS01) on malaria incidence, severity and mortality and determine synergistic effects of combining vaccine with ITNs, IRS and chemoprevention strategies
Animal surveillance data integration	Develop a centralised animal health information system for Kenya tracking outbreak frequency, priority diseases, transmission chains and key outbreak metrics (cases, deaths, risk, location)
Workforce estimation	Develop a proof-of-concept county-level dashboard for HIV by combining disease burden with healthcare human time-resource needs to identify the extent of unmet healthcare human resource needs based on currently deployed human resources

IRS, Indoor residual spraying; ITN, Insecticide-treated nets; RTS,S/AS01, Recombinant circumsporozoite protein-based malaria vaccine with AS01 adjuvant; SMC, Seasonal malaria chemoprevention.

While the generated solutions reached the prototype or proof-of-concept stage, their integration into national health information systems or decision-making processes requires further technical development and institutional alignment. Evaluation of their policy relevance and impact will be undertaken as part of subsequent implementation and dissemination phases.

### Reflections and recommendations

This section synthesises the main lessons from the hackathon, what worked well, what proved challenging and what aspects appear generalisable for future knowledge-translation initiatives.

Participants were asked to provide both positive and negative feedback on their hackathon experience. Semantic analysis was used to identify common themes for the aspects of the hackathon that were perceived to be conducive to an effective hackathon and which, on reflection, could be improved. The following captures the major themes identified, with supporting quotes from participants.

#### Preliminary planning and environment

Having challenge ideas prepared in advance significantly streamlined the hackathon process, allowing participants to dive directly into problem-solving. This approach allowed participants to focus their energy on execution rather than spending valuable hackathon time ideating from scratch.

The challenges were clearly defined, making it easy to hit the ground running. (Lilith Whittles, ICL, facilitator)

The venue choice played a significant role in shaping the hackathon experience. Due to unforeseen circumstances, the event was relocated a few days prior to the hackathon to an alternative nearby venue, which still provided convenient access for both facilitators who had travelled from different regions and participants commuting daily. While the space was functional, it lacked natural light and an open atmosphere, making it more challenging to maintain energy levels and foster creativity.

The venue was good, but a room with daylight would be extremely beneficial to the state of the participants. (Sabine van Elsland, ICL, facilitator)

#### Family dynamics and icebreakers

Creating a welcoming and inclusive environment was key to fostering effective collaboration. Icebreaker activities helped participants overcome initial social barriers, encouraging networking and comfort within families. These activities played an important role in ensuring that individuals from diverse backgrounds and roles in organisations could quickly find common ground, work cohesively and break down hierarchical structures in families.

Through playing jenga, we were able to interact with each other and also got to know more about our colleagues (family). (Decision-maker)

The diversity of participants was a major asset and the most frequently mentioned aspect positively reflected on by participants. With individuals from different professional and cultural backgrounds working together, traditional hierarchies dissolved, allowing for more open and inclusive discussions. This enabled participants to contribute meaningfully regardless of their prior experience.

Being paired up with people of different backgrounds and skills was useful for the cohesion of the team. (Tabitha Okech, CEMA, hacker - modelling focus)

#### Decision-maker involvement

An important aspect of the hackathon was the presence of decision-makers throughout the process. Their engagement provided families with continuous feedback and helped tailor solutions to real-world needs. Having stakeholders present ensured that projects remained aligned with practical applications, increasing the likelihood of real-world implementation.

The family groups that were created grouping the hackers (software engineers, data scientists) and even the policymakers in order to create solutions that are tailored to capture the reality of the problem we are solving. (George Kamundia, CEMA, hacker - software engineer)

However, some families were not joined by decision-makers throughout. Clearer scheduling and communication regarding when decision-makers would be available would have allowed families to optimise their engagement and refine their projects accordingly.

Our group did not really have a decision-maker present, which made it difficult to have a clear idea of what would be most useful for the MoH. (Hacker - software engineer)

#### Facilitation and workflow

Structured facilitation played a crucial role in maintaining momentum throughout the event. Daily debriefs at the end of each day provided participants with an opportunity to reflect on their progress, set priorities and mentally prepare for the next steps. Beginning each day with a short review of previous achievements also helped maintain alignment and motivation among families.

**Figure 2:**
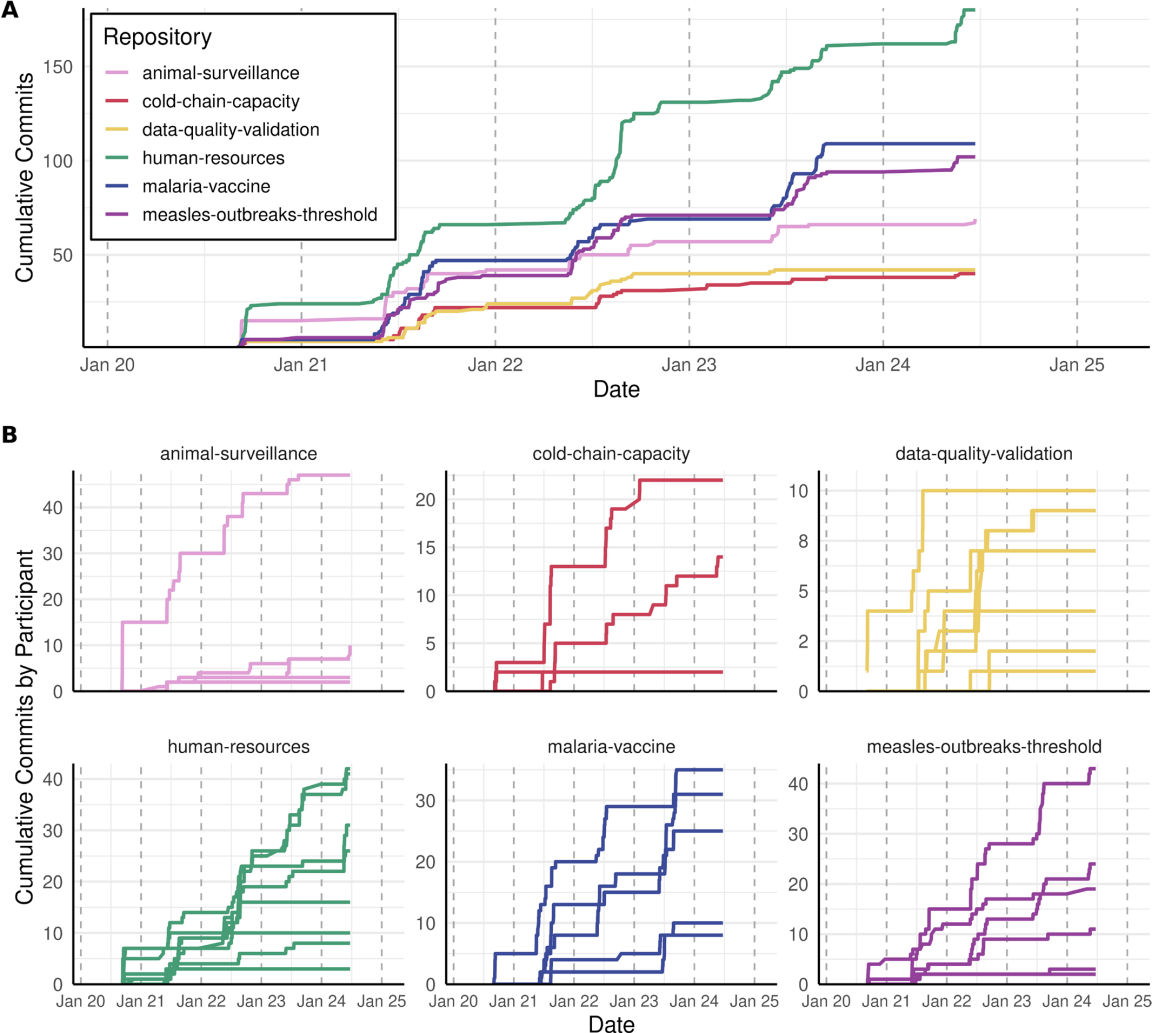
Cumulative GitHub commits by project family. In (A), the cumulative number of commits to the main branch is shown for each project’s GitHub repository and in (B), the cumulative number of commits by each hackathon participant is shown for each repository.

Training on using GitHub was both positively and negatively reflected on, with participants adding that it was helpful to ensure collaboration; however, the different approaches demonstrated for interacting with Git proved confusing. A simple introductory exercise - submitting a pull request to add their name to the README file for their family’s GitHub repository - proved to be an effective way of familiarising participants with GitHub workflows while fostering a sense of contribution early on. This was reinforced during the closing updates each day of the hackathon, in which the number of GitHub commits made by each family was shared (figure 2).

Having debriefs at the end of each day to get to know the progress we've made was an effective component of the hackathon. (Decision-maker)

#### Sustainability and long-term impact

Long-term sustainability remains a critical area for consideration. One potential improvement is assigning facilitator roles to in-house or in-country participants to ensure continued project ownership. Establishing clear norms for remote collaboration and dedicating time to roadmap development would further contribute to sustainable outcomes.

Ideally, I would suggest ‘front-loading’ the idea, code, and other generation tasks so that, by the end of the week, the individuals driving the project are those who will lead it moving forward. In some hackathons, a significant amount of code is written by contributors who cannot continue working on the project. While this is fine if the code is accessible, producing well-commented, accessible code within a short time frame can be a challenge. (Katy Gaythorpe, ICL, facilitator)

#### Unexpected successes and observations

A particular observation from the hackathon was how seamlessly participants worked together. Whether due to the careful selection of participants, strategic family structuring or the energy fostered through effective engagement, families displayed remarkable synergy. Many participants noted that they felt highly motivated and connected, leading to effective collaboration.

Team formation - this was very effective since each group was allocated a diverse set of people with different skill sets. The icebreaker block game - I got to know my team members at a deep level and got to learn some of the things that they love doing. (Kibet Immanuel, 20Four7Va, hacker - software engineering focus)

#### Areas for improvement

Future hackathons would benefit from several adjustments. Providing coding training sessions for policymakers would bridge the technical gap. Allocating more time for testing and refining solutions could result in more sustainable and impactful outcomes. Additionally, inviting topic experts for short talks could deepen both technical and contextual understanding.

In the future, it could be helpful to have more time for testing and refining the final solutions. This will allow the participants to see the project through on to a higher level of innovation. (Preston Osoro, Finesse, hacker - software engineer)

Technical challenges, particularly audiovisual issues, were disruptive. Pretesting equipment and ensuring compatibility across different systems (eg, Mac vs Windows screen resolutions) would smooth presentations. Similarly, access to shared technical resources such as external monitors would facilitate better collaboration.

#### What worked well

The structured 10-stage design, early involvement of decision-makers and daily debriefs were consistently identified as facilitators of productivity and engagement. These elements can be directly transferred to other knowledge translation formats aiming to combine technical co-development with stakeholder ownership.

#### Challenges and generalisable lessons

Variation in participants’ technical skills, limited decision-maker availability and tight timelines were recurrent constraints. Future hackathons may benefit from additional pre-event technical preparation, clearer scheduling for policy participants and post-event mechanisms to support continuity of prototype development. Such adjustments can strengthen hackathons’ utility as scalable knowledge translation platforms.

**Figure 3:**
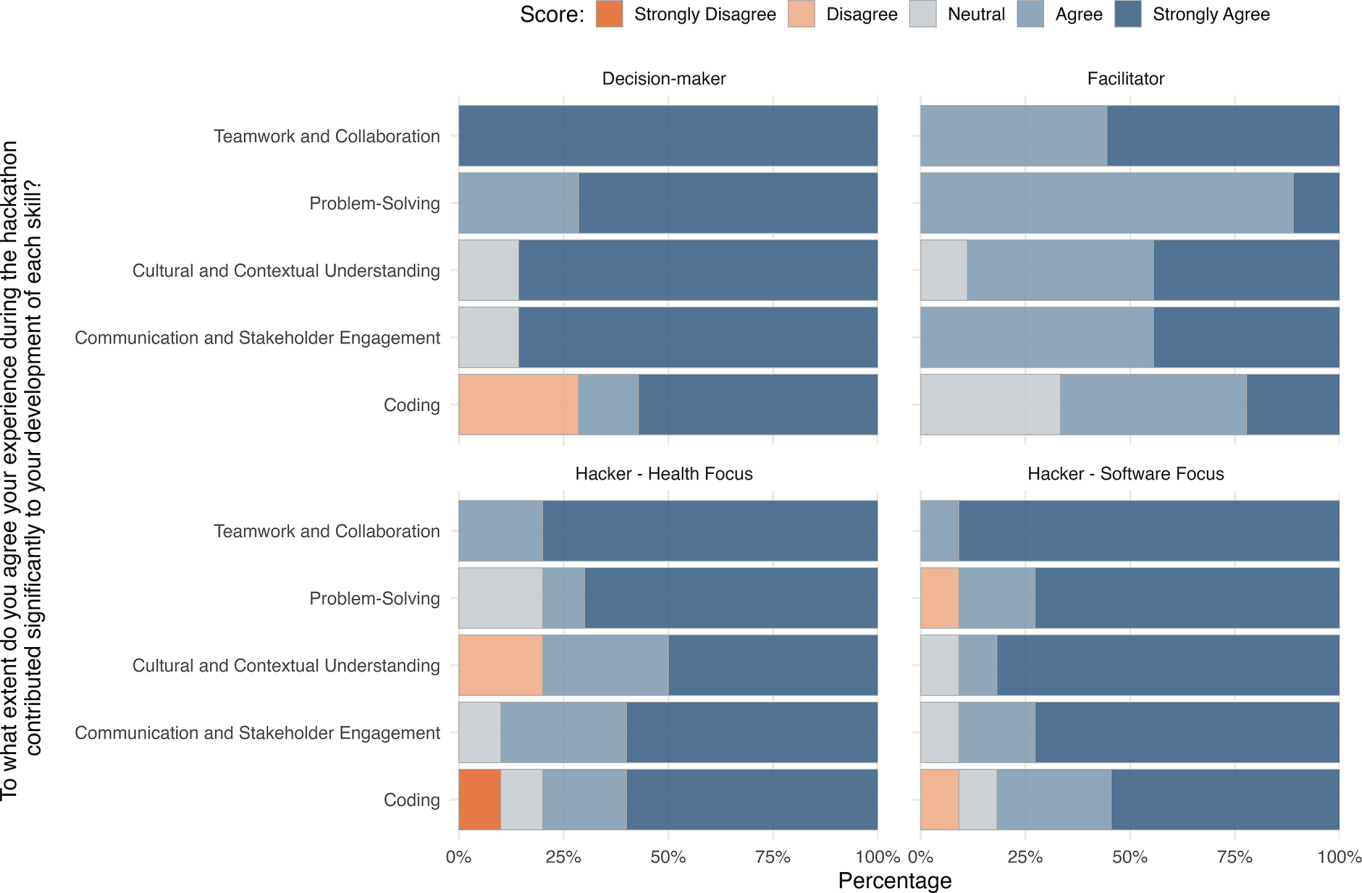
Perceived skill development during the hackathon by participant role. The percentage of scores provided by participants in response to a feedback questionnaire after the hackathon, asking about how the hackathon contributed to the development of five skills (teamwork and collaboration, problem-solving, cultural and contextual understanding, communication and stakeholder engagement, and coding). Responses are displayed on a Likert scale, categorised as strongly disagree, disagree, neutral, agree and strongly agree, and grouped based on participant roles (decision-maker, facilitator, hacker-health focus, hacker-software focus). The colour gradient indicates the Likert scores given, with orange shades representing disagreement and shades of blue representing agreement.

To better prepare families for the final presentations, mentorship in presentation skills and structured templates would be beneficial. Additionally, clearer communication regarding decision-maker availability would help families manage their time more effectively in developing their final presentations. Finally, while quantitative feedback on how participants felt the hackathon improved their skills was consistently positive (figure 3), the different levels of coding experience were remarked on, with some participants feeling more or less time should have been allocated to collaborative coding training. Improving the pre-hackathon material and encouraging participants to undertake self-directed learning before the hackathon would have enabled this training to be shorter. This would have also allowed more time for families to work on their projects, which was a frequent request from participants in feedback.

While the hackathon successfully convened a diverse group of participants, including 83% Kenyan nationals, we recognise that the composition may still have reflected selection biases toward individuals affiliated with well-resourced, urban institutions. As participant data were not collected on sub-national origin, disability or caregiving status, we were unable to formally assess inclusivity across these dimensions. This limits our ability to evaluate the event through a comprehensive equity lens. Future iterations should strengthen outreach to under-represented groups, particularly county-level health officers, rural practitioners and community-based organisations, and include data collection mechanisms to better capture diversity and accessibility considerations. These steps would enhance both the inclusiveness and generalisability of participatory knowledge translation models such as this one.

## Conclusions

Although this paper does not assess the long-term impact of the solutions developed, it provides a foundation for future evaluation. The prototypes and collaborative relationships established during the hackathon continue to evolve through follow-up work between CEMA, Imperial College London and Kenyan public health authorities. Assessing the sustainability and policy adoption of such outputs will be critical to fully understanding the effectiveness of hackathons as tools for knowledge translation.

The BTGH demonstrated the potential of hackathons as a powerful mechanism for bridging the divide between public health research, policy and practice. By fostering interdisciplinary collaboration, real-time problem-solving and stakeholder engagement, the event enabled the co-creation of actionable, data-driven solutions to public health challenges. The experience highlighted the value of structured, participatory approaches to enable knowledge translation, emphasising the importance of sustained engagement, capacity building and iterative solution development. The authors and organisers of the hackathon hope that the experiences shared and lessons learnt here will inspire similar events in the future, with these events holding great potential to innovate how public health projects and partnerships are developed and led in-country. We are also happy to share our experiences further and discuss with individuals considering their own similar events, encouraging others to adapt our approach and resources available through the hackathon website for their own needs.

## Supplementary material

10.1136/bmjgh-2025-019587 online supplemental file 1

## Data Availability

Data sharing not applicable as no datasets generated and/or analysed for this study.
