# Supplementary material for: Bridging the gap between public health, academia and policy
Source: BMJ Glob Health. 2026 Jun 22;11(6):e019587. doi: 10.1136/bmjgh-2025-019587 (PMC13288695; doi:10.1136/bmjgh-2025-019587)
Supplement: online supplemental file 1 [file bmjgh-11-6-s001.docx]

**Supplemental Material**

**Supplemental Figure 1. Jenga as an icebreaker activity.**


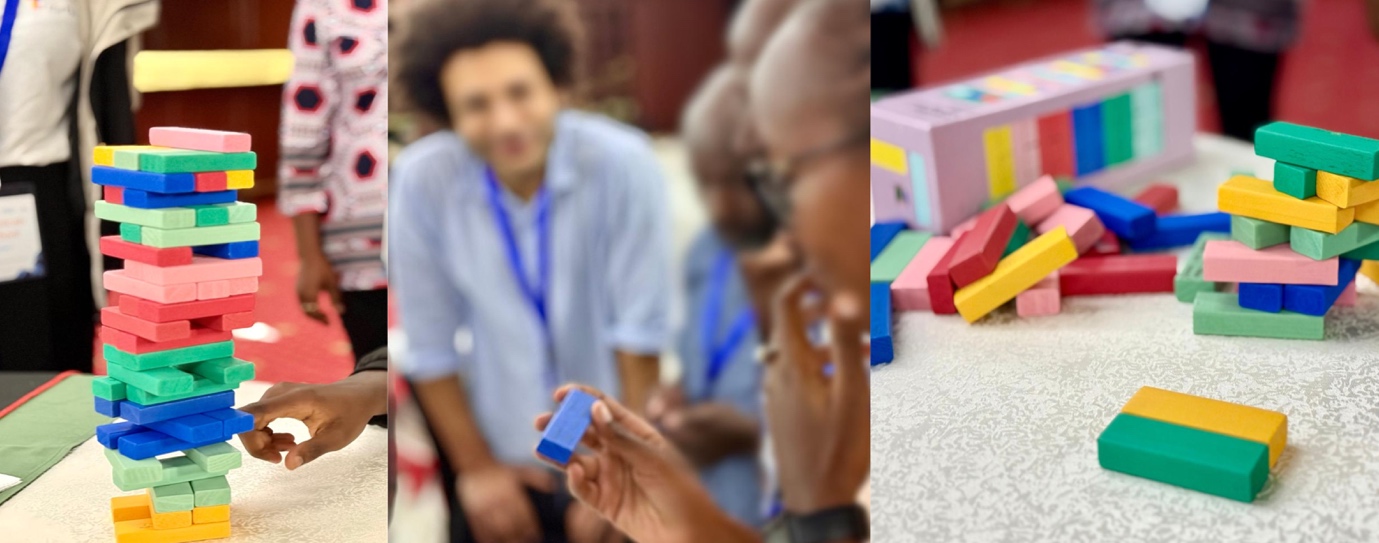


**Supplemental Figure 2. Skills mirror.** Participants’ skills and challenge preferences collected to match them to their families.

**
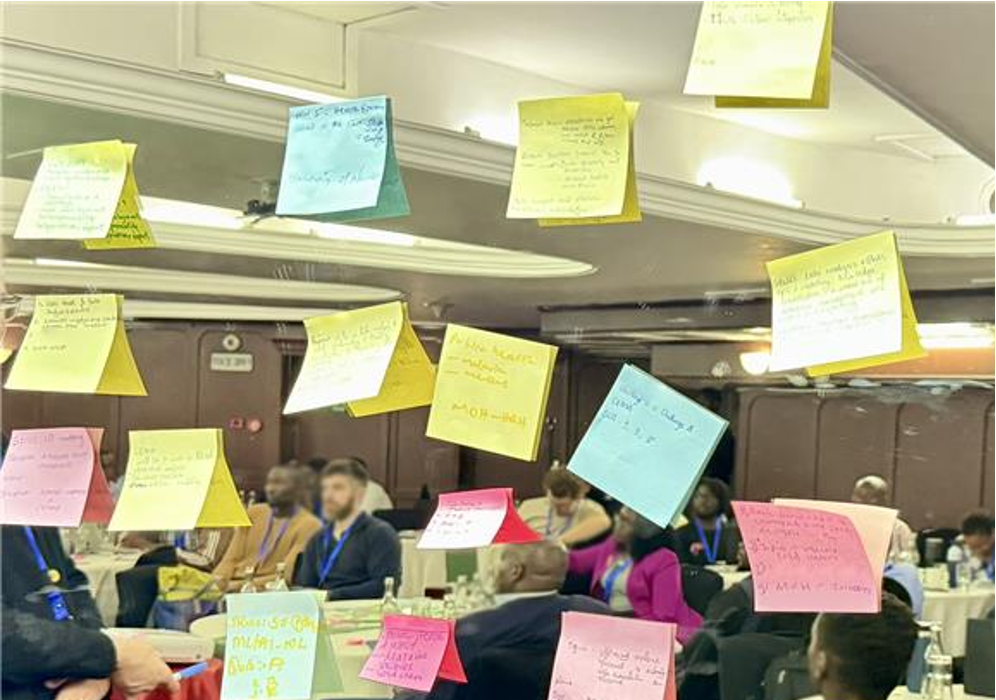
**

**Figure 3. Hackathon Participants.** Breakdown of hackathon participants by **A)** declared gender and ethnicity and **B)** hackathon role**.**

**
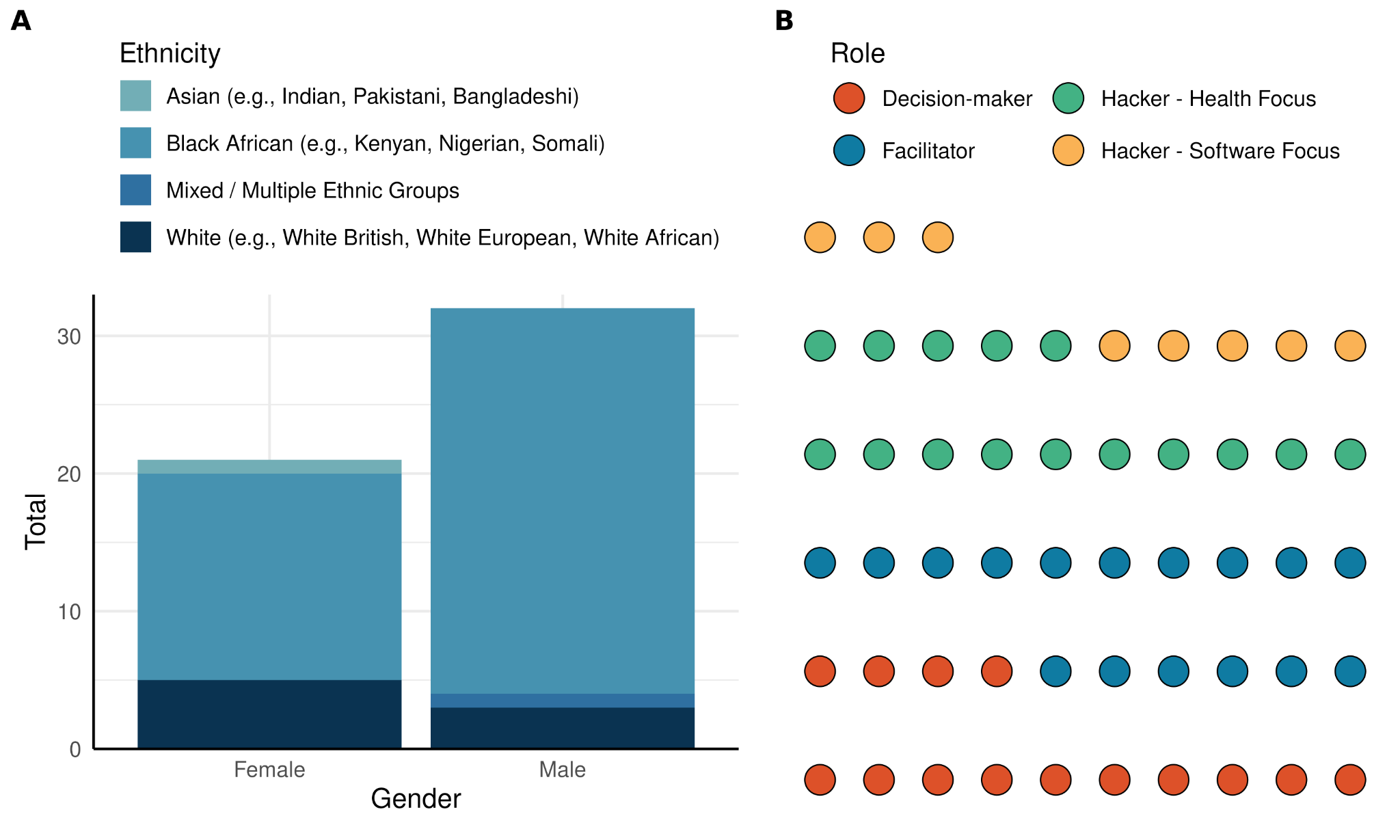
**
